# Cyclophosphamide-induced posterior reversible encephalopathy syndrome (PRES): a case report

**DOI:** 10.1186/1752-1947-8-442

**Published:** 2014-12-18

**Authors:** Jayamalee L Jayaweera, Milinda R Withana, Chamila KP Dalpatadu, Chathurika D Beligaswatta, Thamara Rajapakse, Saroj Jayasinghe, Thashi Chang

**Affiliations:** 47 University Medical Unit, National Hospital, Colombo, Sri Lanka; 48 Department of Radiology, Neuro-Trauma Centre, National Hospital, Colombo, Sri Lanka; 49 Department of Clinical Medicine, Faculty of Medicine, University of Colombo, Colombo, Sri Lanka; 50 8th Mile post, Singheraja Garden, Mathugama, Yattapatha Sri Lanka

**Keywords:** Cyclophosphamide, Posterior reversible encephalopathy syndrome (PRES), Systemic lupus erythematosus

## Abstract

**Introduction:**

Posterior reversible encephalopathy syndrome is a clinicoradiologic entity characterized by headache, seizures, decreased vision, impaired consciousness and white matter oedema in bilateral occipitoparietal regions. Hypertensive encephalopathy, eclampsia, immunosuppressive/cytotoxic drugs, organ transplantation, renal disease, autoimmune diseases and vasculitides are reported risk factors of posterior reversible encephalopathy syndrome. Reports of cyclophosphamide-induced posterior reversible encephalopathy syndrome are rare and occurred in a background of renal failure, fluid overload or active connective tissue disease.

**Case presentation:**

We report a case of posterior reversible encephalopathy syndrome developing as a direct consequence of intravenous cyclophosphamide therapy in a 33-year-old normotensive Sri Lankan woman with lupus nephritis but quiescent disease activity and normal renal function.

**Conclusions:**

This case report highlights the need for awareness and early recognition of this rare but serious adverse effect of cyclophosphamide that occurred in the absence of other known risk factors of posterior reversible encephalopathy syndrome and that early appropriate intervention leads to a good outcome.

## Introduction

Posterior reversible encephalopathy syndrome (PRES) is a clinicoradiologic entity characterized by headache, changes of sensorium, seizures, visual disturbances and vasogenic oedema on neuroimaging [1]. A possible link between autoimmune disorders and PRES has been recently hypothesized [2, 3]. A susceptibility of the cerebrovascular system to insults such as inflammation, hypertension, nephritis, and cytotoxic drugs has been recognized [3, 4]. Cyclophosphamide is an alkylating agent that is widely used in the treatment of selected malignant processes and autoimmune diseases. However, reports of cyclophosphamide-induced PRES are rare [5, 6]. We report the case of a 33-year-old normotensive patient with lupus nephritis who developed PRES following intravenous cyclophosphamide therapy.

## Case presentation

A 33-year-old Sri Lankan woman presented with intermittent inflammatory polyarthritis and low-grade fever of 2 months. She had a childhood history of nephritic syndrome but never had seizures, psychiatric illness or hypertension. On examination, she was pale, had generalized oedema and active non-deforming arthritis involving small and large joints. Urine analysis showed albuminuria, 2 to 3 red blood cells (RBC) per high-power field and RBC casts. Autoimmune screening was positive for antinuclear antibodies (titre >1:160), anti-double-stranded DNA, and a low complement C3 level of 39.3mg/dL (normal range: 55 to 120) and a C4 level of 10.1mg/dL (normal range: 20 to 50). A renal biopsy showed World Health Organization class IV lupus nephritis. Based on a diagnosis of active lupus nephritis, she was treated with 1000mg (15mg**/**kg**/**day) of intravenous methylprednisolone for 3 days and 500mg of intravenous cyclophosphamide. She was discharged from hospital on a maintenance dose of oral prednisolone of 1mg**/**kg**/**day.

Two weeks later, she was readmitted for her second dose of intravenous cyclophosphamide in keeping with the treatment guidelines of the Euro-Lupus Nephritis Trial [7]. On admission, she was clinically well with normal blood counts and inflammatory markers, and a blood pressure of 115/70mmHg. Four hours after completion of the cyclophosphamide infusion she developed generalized tonic–clonic seizures (GTCS). In the next 6 hours, she developed eight GTCS and her level of consciousness deteriorated to a Glasgow Coma Scale (GCS) score of 7/15. She was noted to have a downward gaze with equally reacting pupils and bilaterally extensor plantar reflexes. She was afebrile and did not have neck stiffness. Her blood pressure remained within normal limits. Her haematological and biochemical parameters were within normal limits (haemoglobin 11.8g/dL; white cell count of 9400/ mm^3^; platelet count 236,000/mm^3^; normal blood picture; blood glucose 101mg/dL; serum sodium 141mmol/L; potassium 4.3mmol/L; serum creatinine 99μmol/L; serum ionized calcium 2.2mmol/L; serum magnesium 1.1mmol/L). Her blood and urine cultures were sterile. Her urine analysis was normal except for proteinuria of 30mg/dL. Her erythrocyte sedimentation rate was 18mm/hour, C-reactive protein was 3.3mg/dL, and her complement levels (both C3 and C4) remained within normal limits.

She was electively ventilated. Seizure control was obtained with intravenous midazolam and phenytoin with concurrent nasogastrically delivered high-dose sodium valproate and topiramate.

**Figure 1:**
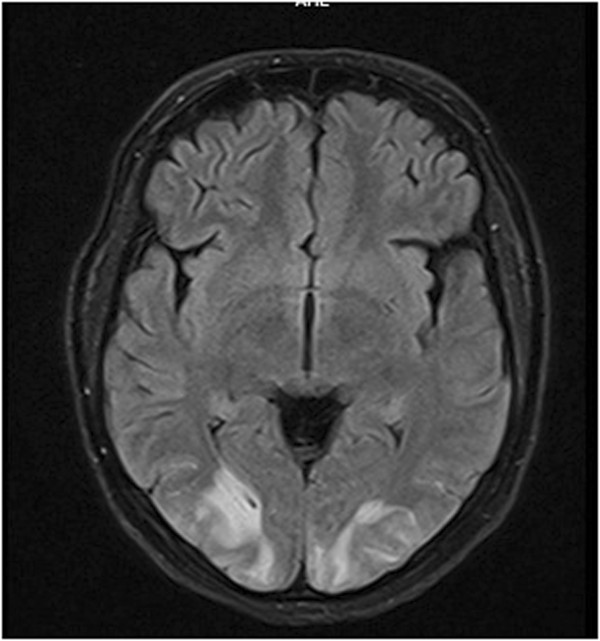
**Magnetic resonance fluid-attenuated inversion recovery image demonstrating symmetrical, hyper intense, white matter lesions in bilateral posterior occipital regions.**

**Figure 2:**
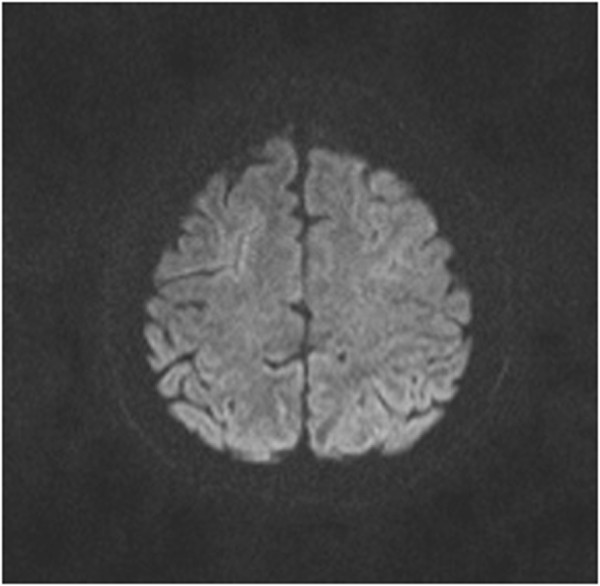
**Diffusion-weighted axial magnetic resonance imaging brain image showing no abnormalities.**

Magnetic resonance imaging of her brain showed symmetrical high-intensity signals confined to the white matter in both occipital regions (Figure 1) with no diffusion restriction (Figure 2) or contrast enhancement compatible with PRES. Magnetic resonance angio- and venograms were normal.

Her level of consciousness improved over the ensuing 24 hours and she was weaned off the ventilator after 48 hours. Her GCS recovered to 15/15 and she remained seizure-free on oral antiepileptic drugs.

## Discussion

Our case report highlights a rare but serious complication of cyclophosphamide pulse therapy in a patient with recently diagnosed class IV lupus nephritis. Although PRES has been reported in association with systemic lupus erythematosus (SLE) and renal disease [5], given that she had quiescent disease activity (Systemic Lupus Erythematosus Disease Activity Index of 2/101) at the time of developing PRES and that it was temporally related to the administration of the drug, cyclophosphamide was considered the most likely aetiological factor. PRES is generally considered to have a benign outcome but grave consequences have been reported in some case series [8] and it is likely to cause irreversible damage if the inciting agent is not removed early.

The underlying pathophysiology of PRES remains elusive but is thought to be a loss of cerebral vascular autoregulation, probably with endothelial dysfunction, in the setting of an acute rise in systemic blood pressure [1]. Cerebral white matter is composed of loosely packed myelinated nerve fibres that makes it more susceptible to vasogenic oedema. The relative deficiency of vasoconstrictor-adrenergic innervation of the vertebrobasilar system compared to the vessels of the carotid system has been hypothesized to explain the posterior preponderance of PRES. However, PRES lesions have been reported to occur in the anterior circulation territory as well as in normotensive patients [8, 9]. In these cases, breakdown of the blood–brain barrier due to endothelial dysfunction caused by certain drugs or underlying conditions such as fluid overload or renal insufficiency have been postulated [3]. Abnormal endothelial activation, dysfunction and leukocyte tracking have recently been reported to cause cerebral and systemic hypoperfusion, which may be causative factors for PRES in SLE [6, 10]. In general, patients with SLE who developed PRES had a high disease activity index at the time of its occurrence [2, 5, 11].

Cyclophosphamide use has been reported in several cases of PRES, but mostly in combination with other cytotoxic agents for treatment of haematological malignancies [2, 12]. Furthermore, PRES attributed to cyclophosphamide is confounded by the presence of fluid overload, hypertension, and/or renal failure, all of which could contribute to endothelial dysfunction [2, 6]. In contrast, our patient did not have any such inciting factors apart from cyclophosphamide therapy, thus making it the most likely causative factor. Early recognition and appropriate therapy ensured a rapid and complete recovery in our patient. The risk of recurrence of PRES with reuse of cyclophosphamide remains unresolved [5, 12]. If the indication permits then an alternative cytotoxic agent may be used. However, close monitoring for recurrence would be necessary.

## Conclusions

Our case report illustrates the occurrence of PRES following administration of cyclophosphamide in a patient who had no other known risk factors for PRES. It highlights the need for a high index of suspicion and a good outcome related to early recognition and appropriate intervention.

## Consent

Written informed consent was obtained from the patient for publication of this case report and accompanying images. A copy of the written consent is available for review by the Editor-in-Chief of this journal.

## Authors’ information

JJ (MBBS, MD) is a senior registrar in Medicine attached to the University Medical Unit of National Hospital of Sri Lanka. MW (MBBS) is a registrar in Medicine attached to the University Medical Unit of National Hospital of Sri Lanka. CDB is a registrar in Medicine attached to the University Medical Unit of National Hospital of Sri Lanka. CD (MBBS, MD) is a senior registrar in Medicine attached to the University Medical Unit of National Hospital of Sri Lanka. TR (MBBS, MD) is a consultant radiologist attached to Neuro-Trauma Center of National Hospital of Sri Lanka. SJ (MBBS, MD, FRCP) is a consultant physician and Professor in the Department of Clinical Medicine, Faculty of Medicine, University of Colombo. TC (MBBS, MD, FRCP, DPhil) is a consultant Neurologist and senior lecturer in the Department of Clinical Medicine, Faculty of Medicine, University of Colombo.

## Abbreviations

GCS: Glasgow Coma Scale
GTCS: Generalized tonic–clonic seizures
PRES: Posterior reversible encephalopathy syndrome
RBC: Red blood cells
SLE: Systemic lupus erythematosus.
